# Supplementary material for: Strengthening the Evidence for a Causal Link between Type 2 Diabetes Mellitus and Pancreatic Cancer: Insights from Two-Sample and Multivariable Mendelian Randomization
Source: Int J Mol Sci. 2024 Apr 23;25(9):4615. doi: 10.3390/ijms25094615 (PMC11082974; doi:10.3390/ijms25094615)
Supplement: Supplementary file 1 [file ijms-25-04615-s001.zip › Supplementary_Table_S13_STROBE-MR-checklist-fillable_v2.pdf]

Supplementary Table S13. STROBE-MR checklist of recommended items to address in reports of Mendelian randomization studies[1 2]

Item No.	Section		Checklist item	Page No.	Relevant text from manuscript
1	**TITLE and ABSTRACT**		Indicate Mendelian randomization (MR) as the study's design in the title and/or the abstract if that is a main purpose of the study	1	Strengthening the evidence for a causal Link between Type 2 Diabetes Mellitus and Pancreatic Cancer: Insights from Two-Sample and Multivariable Mendelian Randomization
	**INTRODUCTION**				
2	**Background**		Explain the scientific background and rationale for the reported study. What is the exposure? Is a potential causal relationship between exposure and outcome plausible? Justify why MR is a helpful method to address the study question	1-2	Despite numerous observational studies revealing an association between type 2 Diabetes Mellitus (T2DM) and an increased risk of pancreatic cancer (PaCa), the causal relationship between T2DM and PaCa remains controversial in Mendelian randomization (MR) studies. MR studies are important for investigating causal relationships. However, the consistency regarding whether the genetic liability to T2DM is causally related to PaCa remains absent in previous studies.
3	**Objectives**		State specific objectives clearly, including pre-specified causal hypotheses (if any). State that MR is a method that, under specific assumptions, intends to estimate causal effects	1-2	In this study, we employed a two-sample MR approach to evaluate the causal effect between T2DM and PaCa, providing insights into the i the associations of genetic liability to T2DM with PaCa..
	**METHODS**				
4	**Study design and data sources**		Present key elements of the study design early in the article. Consider including a table listing sources of data for all phases of the study. For each data source contributing to the analysis, describe the following:		
		a)	Setting: Describe the study design and the underlying population, if possible. Describe the setting, locations, and relevant dates, including periods of recruitment, exposure, follow-up, and data collection, when available.	9-12	The 2SMR study was used to investigate the causal relationship between T2DM and PaCa. We utilized publicly available summary data from genome-wide association studies (GWAS), including the FinnGen, the UKBB, and two genome-wide association meta-analyses. Our research adhered to the three critical assumptions of MR: (1) a strong association between the genetic instruments and T2DM, (2) no association of these instruments with confounding variables, and (3) the exclusive in-fluence of these instruments on PaCa through T2DM.

b)	Participants: Give the eligibility criteria, and the sources and methods of selection of participants. Report the sample size, and whether any power or sample size calculations were carried out prior to the main analysis	9-12	The GWAS summary data for PaCa used in our study were sourced from the R10 release of the FinnGen Consortium[36,37], including both the FinnGen and UK Bi-obank GWAS summary data. For detailed information on the web browser, please refer to: https://public-metaresults-fg-ukbb.finngen.fi/. In the search browser[37], the phe-notype "Malignant neoplasm of pancreas (excluding all other cancers in controls)" was used, including 1,626 cases and 314,193 controls In FinnGen and 936 cases and 400,294 controls in the UK Biobank. In the FinnGen dataset, PaCa cases were identified using codes from ICD-8, ICD-9, and ICD-10, as well as surgery codes and medication pur-chase codes. In the UK Biobank, PaCa cases were diagnosed using codes from ICD-9 and ICD-10, surgery records, and self-reported information.
c)	Describe measurement, quality control and selection of genetic variants	9-12	The selection of IVs for T2DM in this study was based on two genome-wide asso-ciation meta-analyses. The first, known as the DIAGRAM consortium by Maha-jan et al. , encompassed 74,124 T2DM cases and 824,006 controls of European de-scent. Second, a genome-wide association meta-analysis by Vujkovic et al. in-volved 228,499 T2DM cases and 1,178,783 controls in multi-ancestry. The following criteria were applied for the selection of IVs: (1) Initially, we identified single nucleo-tide polymorphisms (SNPs) from the genome-wide association meta-analyses con-ducted by Mahajan et al. and Vujkovic et al. SNPs identified as replication variants were selected for possessing a smaller p-value (n=899). (2) SNPs that met the genome-wide statistical significance threshold (P < 5 × 10-8) were selected as instru-mental variables for T2DM (n=589). (3) The clumping threshold for linkage disequilib-rium (LD) was set at r2 = 0.2 within a 250-kb window[33], using the 1000 Genomes European panel as the reference. Based on this threshold, 436 SNPs were confirmed as independent. (4): 430 SNPs, 434 SNPs, and 435 SNPs were available in FinnGen, UKBB, and a combination of FinnGen and UKBB datasets, respectively. (5) To mitigate issues related to the orientation of strands, we flipped the reverse strand to the forward strand, harmonized the effect of SNPs on exposure and outcome, and dealt with the palindromic SNPs. Ambiguous palindromic SNPs that exhibited a minor allele fre-quency (MAF) greater than 0.42 were discarded. Finally, 414 SNPs in the FinnGen dataset, 423 SNPs in the UKBB dataset, and 423 SNPs

					in a combined FinnGen and UKBB dataset were selected for comprehensive model analysis. (6) Genetic variants near the FTO gene Genetic variants near the FTO gene were reported to be associated with Body Mass Index (BMI). Therefore, to mitigate potential pleiotropic effects, SNPs in the vicinity of the FTO gene were excluded from our restricted model analysis (n=412 (FinnGen), n=421 (UKBB), n=421(FinnGen+UKBB)). The flowchart of IVs selec-tion is shown in Figure 3.
		d)	For each exposure, outcome, and other relevant variables, describe methods of assessment and diagnostic criteria for diseases	9-12	In our study, the IVW method was designated as the primary approach, while the other four robust methods were employed as complementary methods. We first performed MR analysis with all the above-selected IVs. If the MR-PRESSO global test identified horizontal pleiotropy, the outliers would be eliminated, and the MR-PRESSO analysis would be repeated. In addition to the sensitivity analyses, the MR-Egger regression intercept analysis was conducted to examine horizontal pleiotropy, with a p-value < 0.05 considered as evidence of horizontal pleiotropy. Funnel plots were also created for pleiotropy direction detection, where an asymmetrical or skewed pattern may indicate horizontal pleiotropy is present. Furthermore, heterogeneity was assessed through Cochrane's Q test, where a p-value < 0.05 would be considered an indication of heterogeneity. Moreover, a leave-one-out test was executed, systematically removing each SNP to mitigate the potential heterogeneity and consolidate the stability of the estimated causal effect in our study. Ultimately, the MR Steiger directionality test was employed to ascertain the direction of causality by assessing whether the variance explained in the outcome is less than that in the exposure. Our 2SMR analysis was in accordance with the recommendations provided in the STROBE-MR statement. Detailed information is listed in the supplementary. The flowchart of the 2SMR analysis process is shown in Figure 4.
		e)	Provide details of ethics committee approval and participant informed consent, if relevant	12	All data analyzed in this study were obtained from publicly available GWAS summary datasets. The original GWAS had received approval from the relevant ethics committee. This study did not collect any new data; hence, further ethical approval was not necessary.
5	**Assumptions**		Explicitly state the three core IV assumptions for the main analysis (relevance, independence and exclusion restriction) as well assumptions for any additional or sensitivity analysis	9	Our research adhered to the three critical assumptions of MR[32]: (1) a strong associa-tion between the genetic instruments and T2DM, (2) no association of these instru-

					ments with confounding variables, and (3) the exclusive influence of these instruments on PaCa through T2DM.
6	**Statistical methods: main analysis**		Describe statistical methods and statistics used		
		a)	Describe how quantitative variables were handled in the analyses (i.e., scale, units, model)	11	Numerous MR methods were applied in our 2SMR analysis, including IVW[30] and four other robust methods: the MR-Egger method[31], the WM[32] method, the WMO method[33], and the MR-PRESSO method[34].
		b)	Describe how genetic variants were handled in the analyses and, if applicable, how their weights were selected	9	The selection of IVs for T2DM in this study was based on two genome-wide asso-ciation meta-analyses[30,31]. The first, known as the DIAGRAM consortium by Maha-jan et al. [30], encompassed 74,124 T2DM cases and 824,006 controls of European de-scent. Second, a genome-wide association meta-analysis by Vujkovic et al. [31] in-volved 228,499 T2DM cases and 1,178,783 controls in multi-ancestry. The following criteria were applied for the selection of IVs: (1) Initially, we identified single nucleo-tide polymorphisms (SNPs) from the genome-wide association meta-analyses con-ducted by Mahajan et al. [30] and Vujkovic et al. [31] SNPs identified as replication variants were selected for possessing a smaller p-value (n=899). (2) SNPs that met the genome-wide statistical significance threshold (P < 5 × 10-8) were selected as instru-mental variables for T2DM (n=589). (3) The clumping threshold for linkage disequilib-rium (LD) was set at r2 = 0.2 within a 250-kb window[33], using the 1000 Genomes European panel as the reference. Based on this threshold, 436 SNPs were confirmed as independent. (4): 430 SNPs, 434 SNPs, and 435 SNPs were available in FinnGen, UKBB, and a combination of FinnGen and UKBB datasets, respectively. (5) To mitigate issues related to the orientation of strands, we flipped the reverse strand to the forward strand, harmonized the effect of SNPs on exposure and outcome, and dealt with the palindromic SNPs. Ambiguous palindromic SNPs that exhibited a minor allele fre-quency (MAF) greater than 0.42 were discarded[34]. Finally, 414 SNPs in the FinnGen dataset, 423 SNPs in the UKBB dataset, and 423 SNPs in a combined FinnGen and UKBB dataset were selected for comprehensive model analysis. (6) Genetic variants near the FTO gene Genetic variants near the FTO gene were reported to be associated with Body Mass Index (BMI)[35]. Therefore, to mitigate potential pleiotropic effects, SNPs in the vicinity of the

				FTO gene were excluded from our restricted model analysis (n=412 (FinnGen), n=421 (UKBB), n=421(FinnGen+UKBB)). The flowchart of IVs selec-tion is shown in Figure 3.
	c)	Describe the MR estimator (e.g. two-stage least squares, Wald ratio) and related statistics. Detail the included covariates and, in case of two-sample MR, whether the same covariate set was used for adjustment in the two samples	9	The GWAS summary data for PaCa used in our study were sourced from the R10 release of the FinnGen Consortium[36,37], including both the FinnGen and UK Bi-obank GWAS summary data. For detailed information on the web browser, please refer to: https://public-metaresults-fg-ukbb.finngen.fi/. In the search browser[37], the phe-notype "Malignant neoplasm of pancreas (excluding all other cancers in controls)" was used, including 1,626 cases and 314,193 controls In FinnGen and 936 cases and 400,294 controls in the UK Biobank. In the FinnGen dataset, PaCa cases were identified using codes from ICD-8, ICD-9, and ICD-10, as well as surgery codes and medication pur-chase codes. In the UK Biobank, PaCa cases were diagnosed using codes from ICD-9 and ICD-10, surgery records, and self-reported information.
	d)	Explain how missing data were addressed	9	The selection of IVs for T2DM in this study was based on two genome-wide asso-ciation meta-analyses[30,31]. The first, known as the DIAGRAM consortium by Maha-jan et al. [30], encompassed 74,124 T2DM cases and 824,006 controls of European de-scent. Second, a genome-wide association meta-analysis by Vujkovic et al. [31] in-volved 228,499 T2DM cases and 1,178,783 controls in multi-ancestry. The following criteria were applied for the selection of IVs: (1) Initially, we identified single nucleo-tide polymorphisms (SNPs) from the genome-wide association meta-analyses con-ducted by Mahajan et al. [30] and Vujkovic et al. [31] SNPs identified as replication variants were selected for possessing a smaller p-value (n=899). (2) SNPs that met the genome-wide statistical significance threshold (P < 5 × 10-8) were selected as instru-mental variables for T2DM (n=589). (3) The clumping threshold for linkage disequilib-rium (LD) was set at r2 = 0.2 within a 250-kb window[33], using the 1000 Genomes European panel as the reference. Based on this threshold, 436 SNPs were confirmed as independent. (4): 430 SNPs, 434 SNPs, and 435 SNPs were available in FinnGen, UKBB, and a combination of FinnGen and UKBB datasets, respectively. (5) To mitigate issues related to the orientation of strands, we flipped the reverse strand to the forward strand, harmonized the effect of SNPs on exposure and outcome, and dealt with

				the palindromic SNPs. Ambiguous palindromic SNPs that exhibited a minor allele fre-quency (MAF) greater than 0.42 were discarded[34]. Finally, 414 SNPs in the FinnGen dataset, 423 SNPs in the UKBB dataset, and 423 SNPs in a combined FinnGen and UKBB dataset were selected for comprehensive model analysis. (6) Genetic variants near the FTO gene Genetic variants near the FTO gene were reported to be associated with Body Mass Index (BMI)[35]. Therefore, to mitigate potential pleiotropic effects, SNPs in the vicinity of the FTO gene were excluded from our restricted model analysis (n=412 (FinnGen), n=421 (UKBB), n=421(FinnGen+UKBB)). The flowchart of IVs selec-tion is shown in Figure 3.
		e) If applicable, indicate how multiple testing was addressed	12	To mitigate the effects of pleiotropy and reduce bias due to confounding from obesity, we also conducted multivariable Mendelian Randomization (MR). Multivariable MR[38] can assess the influence of multiple exposures on the same outcome. Thus, we utilized multivariable MR to explore the causal relationship between T2DM and PaCa by adjusting for BMI and waist circumference. Initially, the 436 T2DM IVs through LD clumping were searched on the PhenoScanner[39,40] website. Of these, 45 SNPs were found to have overlapping traits with "Body Mass Index" and "Waist Circumference." After harmonizing the SNPs, 43 SNPs were ultimately selected as IVs for our multivariable MR analysis
7	**Assessment of assumptions**	Describe any methods or prior knowledge used to assess the assumptions or justify their validity	10	To avoid bias from weak instruments in this study, we adopted the proportion of variance explained (PVE) known as R^2 for assessing total strength[38] and the F-statistic for measuring average instrument strength[38]. The PVE in the exposure is explained by the selected genetic variants. Generally, a higher PVE is preferable, as it significantly enhances the effectiveness of a MR analysis[38]. The F-statistic was pro-posed to assess IVs strength[39,40]. A commonly used cutoff value is 10[39,40]; an F-statistic less than 10 indicates weak instruments.<br>In this study, the PVE of 436 SNPs after LD clumping was 41.4%, and the F statistic was 986.35. Both the total and average instrument strengths are considered good.<br>The power calculation for two-sample MR analysis was conducted using an online tool (https://shiny.cnsgenomics.com/mRnd/)[71,72], and the

				outcomes are presented in Supplementary Table S12. The variance explained by the genetic instruments associated with T2DM adopted in FinnGen, UKBB, and combined FinnGen and UKBB studies were 39.16%, 40.18%, and 40.31%.
8	**Sensitivity analyses and additional analyses**	Describe any sensitivity analyses or additional analyses performed (e.g. comparison of effect estimates from different approaches, independent replication, bias analytic techniques, validation of instruments, simulations)	11	We first performed MR analysis with all the above-selected IVs. If the MR-PRESSO global test identified horizontal pleiotropy, the outliers would be eliminated, and the MR-PRESSO analysis would be repeated. In addition to the sensitivity analyses, the MR-Egger regression intercept analysis was conducted to examine horizontal pleiotropy, with a p-value < 0.05 considered as evidence of horizontal pleiotropy. Funnel plots were also created for pleiotropy direction detection, where an asymmetrical or skewed pattern may indicate horizontal pleiotropy is present[31]. Furthermore, heterogeneity was assessed through Cochrane's Q test, where a p-value < 0.05 would be considered an indication of heterogeneity. Moreover, a leave-one-out test was executed, systematically removing each SNP to mitigate the potential heterogeneity and consolidate the stability of the estimated causal effect in our study. Ultimately, the MR Steiger directionality test[36] was employed to ascertain the direction of causality by assessing whether the variance explained in the outcome is less than that in the exposure. Our 2SMR analysis was in accordance with the recommendations provided in the STROBE-MR statement[37]. Detailed information is listed in the supplementary. The flowchart of the 2SMR analysis process is shown in Figure
9	**Software and pre-registration**			
	a)	Name statistical software and package(s), including version and settings used	12	A significance level below 0.05 was considered statistically significant, with statistical significance determined by 95% confidence intervals (95% CI) not including one. In this study, the statistical analyses were conducted using R software[55] (version 4.3.2, R Development Core Team, Vienna, Austria). The TwoSampleMR R package[28] and MR-PRESSO R package[56] were employed for all 2SMR analyses, utilizing functions such as harmonise_data, mr, mr_presso, mr_heterogeneity, mr_pleiotropy_test, mr_singlesnp, mr_leaveoneout, mr_scatter_plot, mr_forest_plot, mr_funnel_plot, mv_multiple and directionality_test [57].

		b) State whether the study protocol and details were pre-registered (as well as when and where)	12	A significance level below 0.05 was considered statistically significant, with statistical significance determined by 95% confidence intervals (95% CI) not including one. In this study, the statistical analyses were conducted using R software[55] (version 4.3.2, R Development Core Team, Vienna, Austria). The TwoSampleMR R package[28] and MR-PRESSO R package[56] were employed for all 2SMR analyses, utilizing functions such as harmonise_data, mr, mr_presso, mr_heterogeneity, mr_pleiotropy_test, mr_singlesnp, mr_leaveoneout, mr_scatter_plot, mr_forest_plot, mr_funnel_plot, mv_multiple and directionality_test [57].
	**RESULTS**			
10	**Descriptive data**			
		a) Report the numbers of individuals at each stage of included studies and reasons for exclusion. Consider use of a flow diagram	3,10	The flowchart of IVs selection is shown in Figure 3.
		b) Report summary statistics for phenotypic exposure(s), outcome(s), and other relevant variables (e.g. means, SDs, proportions)	10	The GWAS summary data for PaCa used in our study were sourced from the R10 release of the FinnGen Consortium[36,37], including both the FinnGen and UK Bi-obank GWAS summary data. For detailed information on the web browser, please refer to: https://public-metaresults-fg-ukbb.finngen.fi/. In the search browser[37], the phe-notype "Malignant neoplasm of pancreas (excluding all other cancers in controls)" was used, including 1,626 cases and 314,193 controls In FinnGen and 936 cases and 400,294 controls in the UK Biobank. In the FinnGen dataset, PaCa cases were identified using codes from ICD-8, ICD-9, and ICD-10, as well as surgery codes and medication pur-chase codes. In the UK Biobank, PaCa cases were diagnosed using codes from ICD-9 and ICD-10, surgery records, and self-reported information.
		c) If the data sources include meta-analyses of previous studies, provide the assessments of heterogeneity across these studies	NA	
		d) For two-sample MR:<br>i. Provide justification of the similarity of the genetic variant-exposure associations between the exposure and outcome samples<br>ii. Provide information on the number of individuals who overlap between the exposure and outcome studies	10	The selection of IVs for T2DM in this study was based on two genome-wide asso-ciation meta-analyses[30,31]. The first, known as the DIAGRAM consortium by Maha-jan et al. [30], encompassed 74,124 T2DM cases and 824,006 controls of European de-scent. Second, a genome-wide association meta-analysis by Vujkovic et al. [31] in-volved 228,499 T2DM cases and 1,178,783 controls in multi-

				ancestry. The following criteria were applied for the selection of IVs: (1) Initially, we identified single nucleo-tide polymorphisms (SNPs) from the genome-wide association meta-analyses con-ducted by Mahajan et al. [30] and Vujkovic et al. [31] SNPs identified as replication variants were selected for possessing a smaller p-value (n=899). (2) SNPs that met the genome-wide statistical significance threshold (P < 5 × 10-8) were selected as instru-mental variables for T2DM (n=589). (3) The clumping threshold for linkage disequilib-rium (LD) was set at r2 = 0.2 within a 250-kb window[33], using the 1000 Genomes European panel as the reference. Based on this threshold, 436 SNPs were confirmed as independent. (4): 430 SNPs, 434 SNPs, and 435 SNPs were available in FinnGen, UKBB, and a combination of FinnGen and UKBB datasets, respectively. (5) To mitigate issues related to the orientation of strands, we flipped the reverse strand to the forward strand, harmonized the effect of SNPs on exposure and outcome, and dealt with the palindromic SNPs. Ambiguous palindromic SNPs that exhibited a minor allele fre-quency (MAF) greater than 0.42 were discarded[34]. Finally, 414 SNPs in the FinnGen dataset, 423 SNPs in the UKBB dataset, and 423 SNPs in a combined FinnGen and UKBB dataset were selected for comprehensive model analysis. (6) Genetic variants near the FTO gene Genetic variants near the FTO gene were reported to be associated with Body Mass Index (BMI)[35]. Therefore, to mitigate potential pleiotropic effects, SNPs in the vicinity of the FTO gene were excluded from our restricted model analysis (n=412 (FinnGen), n=421 (UKBB), n=421(FinnGen+UKBB)). The flowchart of IVs selec-tion is shown in Figure 3.
11	**Main results**			
		a) Report the associations between genetic variant and exposure, and between genetic variant and outcome, preferably on an interpretable scale	3	In the FinnGen dataset, a causal association between T2DM and PaCa risk was in-dicated by both the IVW (p=0.033) and MR-PRESSO (p=0.029) methods (Table 1). For a one-unit increase in the log-transformed odds of T2DM, the OR of PaCa risk was esti-mated at 1.102 (95% CI= 1.008-1.204) by the IVW method, and 1.097 (95% CI= 1.010-1.191) by the MR-PRESSO method, respectively (Table 1). The MR-PRESSO glob-al test identified two outlier SNPs; consequently, these outliers were corrected in our MR-PRESSO analysis. After eliminating two SNPs near the FTO gene in the restricted model, the causal link between T2DM and PaCa risk

			remained significant in both the IVW (p=0.046) and MR-PRESSO (p=0.031) methods (Table 1) in the FinnGen dataset. With each unit increment in the log-transformed odds of T2DM, the OR for PaCa risk was 1.095 (95% CI= 1.001-1.198) and 1.094 (95% CI=1.008-1.187) via the IVW method and MR-PRESSO method, respectively. Five outlier SNPs were detected in the MR-PRESSO global test; therefore, these outliers were removed in our MR-PRESSO analysis.
b)	Report MR estimates of the relationship between exposure and outcome, and the measures of uncertainty from the MR analysis, on an interpretable scale, such as odds ratio or relative risk per SD difference	3	Within the UKBB dataset, the WM (p=0.022), IVW (p=0.001), and MR-PRESSO (p=0.005) methods revealed a causal relationship between T2DM and PaCa risk (Table 1). These methods indicated a 23.7% increase (OR=1.237, 95% CI= 1.031-1.482) for WM, an 18.5% increase in the odds of PaCa risk (OR=1.185, 95% CI= 1.068-1.315) for IVW, and a 16.2% increase (OR=1.162, 95% CI=1.045-1.288) for MR-PRESSO, per one-unit increase in the log-transformed odds of T2DM (Table 1). After eliminating two SNPs near the FTO gene in the restricted model, the WM (p=0.041), IVW (p=0.002), and MR-PRESSO (p=0.007) methods persistently demonstrated a causal link between T2DM and PaCa risk, as shown in Table 1. A one-unit rise in the log-transformed odds of T2DM correlated with a 23% increase (OR=1.23, 95% CI= 1.008-1.501) in the WM method, an 18% elevation in PaCa risk odds (OR=1.179, 95% CI= 1.061-1.310) in the IVW method, and a 15.6% rise (OR=1.156, 95% CI=1.042-1.284) in the MR-PRESSO method (Table 1).
c)	If relevant, consider translating estimates of relative risk into absolute risk for a meaningful time period	3	For the combined FinnGen and UKBB dataset, the causal effect of T2DM on PaCa risk was demonstrated significantly using the WM (p=0.017), IVW (p=0.001), and MR-PRESSO (p<0.001) approaches (Table 1). With a one-unit increase in the log-transformed odds of T2DM, the OR of PaCa risk was elevated by 15.1% (OR= 1.151, 95% CI= 1.025-1.293) in the WM approach, 13.1% (OR= 1.131, 95% CI= 1.052-1.216) in the IVW method, and 12.7% (OR= 1.127, 95% CI= 1.056-1.204) in the MR-PRESSO method (Table 1). Two outlier SNPs were detected in the MR-PRESSO global test; therefore, these outliers were removed in our MR-PRESSO analysis. After two SNPs removal in the restricted model, the IVW (p=0.002) and MR-PRESSO (p=0.001) meth-ods still significantly highlighted the causal effect of T2DM on PaCa risk, as shown in Table 1. For each unit increase in the log-transformed odds of T2DM, there was a 12.5%

					increase in PaCa risk odds (OR= 1.125, 95% CI= 1.046-1.211) according to the IVW method, and a 12.4% rise (OR= 1.124, 95% CI= 1.053-1.201) via the MR-PRESSO meth-od. The MR-PRESSO global test detected five outlier SNPs; hence, these outliers were eliminated in our MR-PRESSO analysis.
		d)	Consider plots to visualize results (e.g. forest plot, scatterplot of associations between genetic variants and outcome versus between genetic variants and exposure)	4,5	In the scatter plot (Figure 1), the direction of the causal effect of T2DM on PaCa risk was consistently depicted across all MR analysis approaches in the FinnGen, UKBB, and combined FinnGen and UKBB datasets, both in the comprehensive and re-stricted models
12	**Assessment of assumptions**				
		a)	Report the assessment of the validity of the assumptions	3	In our 2SMR analysis, inverse variance weighted (IVW) method was designated as the principal method due to its higher statistical efficacy. Additionally, four robust methods, including the Mendelian randomization-Egger (MR-Egger), the weighted median (WM), the weighted mode (WMO), and the Mendelian Randomization Pleiot-ropy RESidual Sum and Outlier (MR-PRESSO), were employed as complementary ap-proaches to evaluate the genetic causal associations between T2DM and PaCa risk.
		b)	Report any additional statistics (e.g., assessments of heterogeneity across genetic variants, such as I^2, Q statistic or E-value)	5-6	The MR-Egger regression intercept analysis revealed no horizontal pleiotropy in the FinnGen, UKBB, and combined FinnGen and UKBB datasets, both in the compre-hensive and restricted models (Supplementary Table S4). Furthermore, in our funnel plot visualization (Figure 2), general symmetry suggests the absence of horizontal pleiotropy. In Cochran's Q test for IVW, heterogeneity (p<0.05) was observed in the analyses of the Finn and the combined FinnGen and UKBB datasets. According to the leave-one-out analysis, the sequential removal of each IV did not impact the causal relationship between T2DM and PaCa, nor did it affect the OR. Furthermore, the leave-one-out test did not reveal any potential outliers or evidence of horizontal plei-otropy. The details of the leave-one-out test are listed in Supplementary Table S5-S10. In the MR Steiger directionality test, the variance explained in the outcome is less than that in the exposure, confirming the correct causal direction to be true (Supplementary Table S11).<br>In the multivariable MR analysis, the observed association between T2DM and PaCa remained

					significant after adjusting for BMI and Waist circumference (OR=1.485, p<0.001, 95%CI=1.228-1.796) (Table 2).
13	**Sensitivity analyses and additional analyses**				
		a)	Report any sensitivity analyses to assess the robustness of the main results to violations of the assumptions	5	The MR-Egger regression intercept analysis revealed no horizontal pleiotropy in the FinnGen, UKBB, and combined FinnGen and UKBB datasets, both in the compre-hensive and restricted models (Supplementary Table S4). Furthermore, in our funnel plot visualization (Figure 2), general symmetry suggests the absence of horizontal pleiotropy. In Cochran's Q test for IVW, heterogeneity (p<0.05) was observed in the analyses of the Finn and the combined FinnGen and UKBB datasets. According to the leave-one-out analysis, the sequential removal of each IV did not impact the causal relationship between T2DM and PaCa, nor did it affect the OR. Furthermore, the leave-one-out test did not reveal any potential outliers or evidence of horizontal plei-otropy. The details of the leave-one-out test are listed in Supplementary Table S5-S10. In the MR Steiger directionality test, the variance explained in the outcome is less than that in the exposure, confirming the correct causal direction to be true (Supplementary Table S11).
		b)	Report results from other sensitivity analyses or additional analyses	6	In the multivariable MR analysis, the observed association between T2DM and PaCa remained significant after adjusting for BMI and Waist circumference (OR=1.485, p<0.001, 95%CI=1.228-1.796) (Table 2).
		c)	Report any assessment of direction of causal relationship (e.g., bidirectional MR)	5	In the MR Steiger directionality test, the variance explained in the outcome is less than that in the exposure, confirming the correct causal direction to be true (Supplementary Table S11).
		d)	When relevant, report and compare with estimates from non-MR analyses	5-6	The MR-Egger regression intercept analysis revealed no horizontal pleiotropy in the FinnGen, UKBB, and combined FinnGen and UKBB datasets, both in the compre-hensive and restricted models (Supplementary Table S4). Furthermore, in our funnel plot visualization (Figure 2), general symmetry suggests the absence of horizontal pleiotropy. In Cochran's Q test for IVW, heterogeneity (p<0.05) was observed in the analyses of

				the Finn and the combined FinnGen and UKBB datasets. According to the leave-one-out analysis, the sequential removal of each IV did not impact the causal relationship between T2DM and PaCa, nor did it affect the OR. Furthermore, the leave-one-out test did not reveal any potential outliers or evidence of horizontal plei-otropy. The details of the leave-one-out test are listed in Supplementary Table S5-S10. In the MR Steiger directionality test, the variance explained in the outcome is less than that in the exposure, confirming the correct causal direction to be true (Supplementary Table S11).
		e) Consider additional plots to visualize results (e.g., leave-one-out analyses)	5	The details of the leave-one-out test are listed in Supplementary Table S5-S10.
	**DISCUSSION**			
14	**Key results**	Summarize key results with reference to study objectives	6	To explore the causal relationship between T2DM and PaCa, we performed a 2SMR analysis using two large T2DM genome-wide association meta-analyses[30,31], and PaCa cases from FinnGen and UK Biobank datasets. The IVW method, along with four other robust methods, were utilized in the MR analysis. Sensitivity analyses, MR Steiger directionality test, and multivariable MR, were conducted to strengthen our results. Our findings revealed that genetic liability to T2DM was associated with high-er PaCa risk. Our 2SMR results provided evidence of causal associations between T2DM and PaCa.
15	**Limitations**	Discuss limitations of the study, taking into account the validity of the IV assumptions, other sources of potential bias, and imprecision. Discuss both direction and magnitude of any potential bias and any efforts to address them	8	Nonetheless, some limitations still need to be considered in this study. Firstly, both the IVs and the outcome data were obtained from European population-based datasets. Hence, this limits our findings from being generalized to other populations. Secondly, heterogeneity among IVs was detected in some FinnGen analyses using Cochran's Q test for the IVW method. However, the MR-Egger test and other sensitiv-ity analyses did not indicate pleiotropy. In the MR-PRESSO test, causal inference re-mained significant even after correcting for outliers. Additionally, in the comprehen-sive model, the WM approach also revealed a significant effect in the combined Fin-nGen and UK Biobank analysis. Therefore, the likelihood of bias in the results due to pleiotropic effects or invalid IVs is reduced. Lastly, although disease diagnoses are de-fined by ICD codes and electronic healthcare records, there still exists the possibility of detection bias among

				T2DM and PaCa cases. Nevertheless, our results are derived from two large GWAS meta-analyses<br><br>and two extensive population-based datasets, which may overcome individual errors or biases in disease identification.
16	**Interpretation**			
		a) Meaning: Give a cautious overall interpretation of results in the context of their limitations and in comparison with other studies	7-8	Our findings from the 2SMR study, strengthen the evidence for a causal associa-tion between T2DM and PaCa, and further support previous observational stud-ies[14,15,19,62,63]. In our two previous UK Biobank cohort studies[14,15], the OR of PaCa were 2.08 and 2.57 in participants with a history of DM compared to controls without a history of DM. Additionally, both new-onset and long-term DM have been reported to approximately double the risk of PaCa[19,62,63]. An umbrella review[61] also revealed a pooled OR of roughly 2 for PaCa risk among patients with T2DM com-pared to controls.<br><br>Recognizing the causal link between T2DM and PaCa can raise awareness of tar-geting T2DM for pancreatic cancer prevention. Understanding these pathophysiologi-cal mechanisms could potentially pave the way for developing targeted prevention and treatment strategies.
		b) Mechanism: Discuss underlying biological mechanisms that could drive a potential causal relationship between the investigated exposure and the outcome, and whether the gene-environment equivalence assumption is reasonable. Use causal language carefully, clarifying that IV estimates may provide causal effects only under certain assumptions	7-8	The pathophysiological mechanisms linking T2DM to PaCa are complex and mul-tifaceted, encompassing insulin resistance and hyperinsulinemia[17,64-66], persistent hyperglycemia[67-70], chronic inflammation[17,71,72], alterations in gut microbio-ta[73-77], and dysregulated adipokine secretion[17,78-82]. Insulin resistance in T2DM leads to hyperinsulinemia, potentially promoting tumor growth through directly by acting on insulin receptors or indirectly by increasing levels of insulin-like growth fac-tor-1 (IGF-1), both of which can stimulate cell proliferation and inhibit apoptosis to enhanced cell proliferation pathways[64-66]. Additionally, hyperglycemia provides an energy-rich environment for cancer cells, inducing oxidative stress and leading to DNA damage[67-70]. Concurrently, chronic inflammation associated with T2DM cre-ates a pro-inflammatory environment conducive to pancreatic carcinogenesis[71,72]. Some inflammatory mediators or cytokines, such as interleukin-6 (IL-6) and tumor ne-crosis factor-alpha (TNF-α), can promote tumor growth and metastasis[71,72]. Changes in gut microbiota related

				to T2DM may influence systemic inflammation and metabolic profiles, which may affect cancer development via alterations in bile acid metabolism and the release of metabolites that may have carcinogenic proper-ties[73-77]. Moreover, altered adipokine secretion, characterized by increased leptin and decreased adiponectin levels due to adiposity in T2DM, may facilitate cancer pro-gression through pro-inflammatory and anti-apoptotic effects[78-82]. These intercon-nected pathways underscore the complex relationship between T2DM and PaCa.
		c) Clinical relevance: Discuss whether the results have clinical or public policy relevance, and to what extent they inform effect sizes of possible interventions	7-8	Our findings from the 2SMR study, strengthen the evidence for a causal associa-tion between T2DM and PaCa, and further support previous observational stud-ies[14,15,19,62,63]. In our two previous UK Biobank cohort studies[14,15], the OR of PaCa were 2.08 and 2.57 in participants with a history of DM compared to controls without a history of DM. Additionally, both new-onset and long-term DM have been reported to approximately double the risk of PaCa[19,62,63]. An umbrella review[61] also revealed a pooled OR of roughly 2 for PaCa risk among patients with T2DM com-pared to controls.<br><br>Recognizing the causal link between T2DM and PaCa can raise awareness of tar-geting T2DM for pancreatic cancer prevention. Understanding these pathophysiologi-cal mechanisms could potentially pave the way for developing targeted prevention and treatment strategies.
17	**Generalizability**	Discuss the generalizability of the study results (a) to other populations, (b) across other exposure periods/timings, and (c) across other levels of exposure	12-13	Our findings indicate that a causal relationship between T2DM and PaCa has been established in the 2SMR and multivariable MR studies. This discovery could en-hance awareness and implementation of early prevention and detection strategies for PaCa. These strategies include managing diabetes as a preventive measure against PaCa and emphasizing the importance of controlling blood sugar levels and other metabolic risk factors. Additionally, increasing public awareness of the causal link between T2DM and PaCa could underscore the significance of lifestyle interventions, such as diet habits, physical activity, and weight management, not only for diabetes management but also for reducing the risk of PaCa. Furthermore, understanding the causal pathways could lead to discovering biomarkers and developing pharmacologi-cal strategies to improve treatment outcomes and enable more personalized treatment plans. Therefore, further studies are necessary to elucidate the

				precise pathophysiolog-ical mechanisms involved. Ultimately, it aims to reduce the incidence and mortality associated with PaCa.
	**OTHER INFORMATION**			
18	**Funding**	Describe sources of funding and the role of funders in the present study and, if applicable, sources of funding for the databases and original study or studies on which the present study is based	13	This research was funded by the European Union's funded Project iHelp, grant number 101017441.
19	**Data and data sharing**	Provide the data used to perform all analyses or report where and how the data can be accessed, and reference these sources in the article. Provide the statistical code needed to reproduce the results in the article, or report whether the code is publicly accessible and if so, where	13	The datasets analyzed during the current study are available in the FinnGen repository (https://r10.finngen.fi/). The summary statistics can be accessed by applying at: https://elomake.helsinki.fi/lomakkeet/124935/lomake.html.
20	**Conflicts of Interest**	All authors should declare all potential conflicts of interest	13	The authors declare no conflict of interest. The funders had no role in the design of the study; in the collection, analyses, or interpretation of data; in the writing of the manuscript, or in the decision to publish the results

This checklist is copyrighted by the Equator Network under the Creative Commons Attribution 3.0 Unported (CC BY 3.0) license.

1. Skrivankova VW, Richmond RC, Woolf BAR, Yarmolinsky J, Davies NM, Swanson SA, et al. Strengthening the Reporting of Observational Studies in Epidemiology using Mendelian Randomization (STROBE-MR) Statement. JAMA. 2021;under review.

2. Skrivankova VW, Richmond RC, Woolf BAR, Davies NM, Swanson SA, VanderWeele TJ, et al. Strengthening the Reporting of Observational Studies in Epidemiology using Mendelian Randomisation (STROBE-MR): Explanation and Elaboration. BMJ. 2021;375:n2233.
